# The crystal structure of the Sgt1-Skp1 complex: the link between Hsp90 and both SCF E3 ubiquitin ligases and kinetochores

**DOI:** 10.1038/srep41626

**Published:** 2017-01-31

**Authors:** Oliver Willhoft, Richard Kerr, Dipali Patel, Wenjuan Zhang, Caezar Al-Jassar, Tina Daviter, Stefan H. Millson, Konstantinos Thalassinos, Cara K. Vaughan

**Affiliations:** 1 Institute of Structural and Molecular Biology, University College London and Birkbeck, Biological Sciences, Malet Street, London, WC1E 7HX, UK; 2 Institute of Structural and Molecular Biology, University College London and Birkbeck, Division of Biosciences, Darwin Building, Gower Street, London, WC1E 6BT, UK; 3 School of Life Sciences, Joseph Banks Laboratory, University of Lincoln, Lincoln, LN6 7TS, UK

## Abstract

The essential cochaperone Sgt1 recruits Hsp90 chaperone activity to a range of cellular factors including SCF E3 ubiquitin ligases and the kinetochore in eukaryotes. In these pathways Sgt1 interacts with Skp1, a small protein that heterodimerizes with proteins containing the F-box motif. We have determined the crystal structure of the interacting domains of *Saccharomyces cerevisiae* Sgt1 and Skp1 at 2.8 Å resolution and validated the interface in the context of the full-length proteins in solution. The BTB/POZ domain of Skp1 associates with Sgt1 via the concave surface of its TPR domain using residues that are conserved in humans. Dimerization of yeast Sgt1 occurs via an insertion that is absent from monomeric human Sgt1. We identify point mutations that disrupt dimerization and Skp1 binding *in vitro* and find that the interaction with Skp1 is an essential function of Sgt1 in yeast. Our data provide a structural rationale for understanding the phenotypes of temperature-sensitive Sgt1 mutants and for linking Skp1-associated proteins to Hsp90-dependent pathways.

Proteostasis is critical for the regulation of normal cellular function and its loss is frequently a precursor of disease states. Molecular chaperones and their accessory proteins play a crucial role in proteostasis as they enable protein folding and activation, as well as direct proteins for degradation via the ubiquitin-proteasome pathway.

Sgt1 is an essential conserved cochaperone of the molecular chaperone Hsp90. It is unique amongst Hsp90 cochaperones in that it is has been linked genetically and biochemically to both folding and degradation pathways. It was first identified via genetic screens as a protein that directly interacts with the small adapter protein Skp1, in both the Skp1/Cullin/F-box (SCF) E3 ubiquitin ligase pathway, and in assembly of the essential *Saccharomyces cerevisiae* inner kinetochore complex, Centromere Binding Factor 3 (CBF3)^1^ (Fig. 1A). These functionally distinct pathways share a degree of structural homology: a common feature of both systems is the interaction of Skp1 with proteins comprising an F-box motif followed by a Leucine Rich Repeat domain (LRR), known as FBXL proteins. In a subset of SCF E3 ubiquitin ligases (hereafter referred to as SCFs), the complex between Skp1 and the FBXL protein provides specificity for the substrate to be ubiquitylated^2^,^3^. In the yeast kinetochore CBF3 complex of the yeast kinetochore, Skp1 forms a heterodimer with the FBXL protein, Ctf13^4^.

Sgt1 has also been implicated in the innate immune response in humans and disease resistance in plants. In the former the Nod-like receptors (NLRs), NALP3, Nod1 and Nod2, all known effectors of the human inflammatory response, are dependent on Sgt1 activity^5^,^6^, whereas in the latter a wide range of disease resistance ‘R’ proteins require Sgt1 function^7^,^8^. Sgt1 is also required for sensing the plant hormones auxin and jasmonic acid, which are effectors of temperature regulation, growth and disease resistance via the TIR1^9^ and COI1 SCFs^10^. Both TIR1 and COI1 have recently been shown to be clients for the Hsp90-Sgt1 chaperone complex^11^,^12^. In addition Sgt1 is required for the activation of adenylyl cyclase, Cyr1, in *S. cerevisiae* and this direct interaction regulates morphogenesis and drug-resistance in *Candida albicans*^13^.

Recent studies have found that Sgt1 is overexpressed in several cancers, including breast, lung and colon cancer, with Sgt1 controlling the stability of the phosphatase PHLPP1 in gastric tumor cells^14^,^15^,^16^.

In each case these Sgt1-dependent proteins contain an LRR domain which suggest that it is specifically this fold that is targeted by Sgt1^17^. This hypothesis is supported by a cochaperone interactome analysis that identified LRR-domains are the predominant interactors of Sgt1^18^.

Sgt1 comprises 3 domains connected by variable linkers (Fig. 1B). The N-terminal domain of Sgt1 is predicted to have a helical TPR fold (Tetratrico-Peptide Repeat) and is responsible for the interaction with Skp1^19^,^20^. It is also the site of Sgt1 dimerization in *S. cerevisiae* and plants^21^,^22^. By contrast human Sgt1 is monomeric *in vitro*^21^. The site of interaction with Hsp90 is through the middle domain which adopts a CS fold (Chord-containing proteins and Sgt1). This is linked to the TPR domain by a Variable Region (VR). The structure of the CS domain has been solved, both alone by NMR^23^ and in complex with the N-terminal domain of Hsp90 by crystallography^24^. This Hsp90 interaction appears to have different nucleotide dependencies in different organisms. In humans and *Caenorhabditis elegans* Sgt1 has a higher affinity for Hsp90 in the ATP- or ATPγS-bound state, while in yeast Sgt1 the affinity for Hsp90 is higher in the apo and ADP-bound states^20^,^23^,^25^. Lastly, the highly conserved and largely unstructured C-terminal SGS (Sgt1-specific) domain provides an Hsp70 binding site^12^,^26^ as well as the putative site of interaction with proteins containing the LRR fold^17^. It is also the site of regulatory phosphorylation by CK2 in yeast and mutation of serine 361 to the phospho-mimetic aspartic acid is lethal^27^,^28^. This site is conserved in humans although Polo-like kinase 1 has been identified as the relevant kinase in place of CK2^28^.

The structure of the adaptor Skp1 and its function as a component of the SCF complexes has been well-characterised^29^,^30^. Skp1 has an N-terminal BTB/POZ domain^31^ that interacts with Cullin1, a structural subunit of the SCF complexes that arranges the substrate and ligase subunits in close proximity, and a C-terminal extension of around 35 residues, that folds around the helices of F-box motifs (Fig. 1B)^32^. The Skp1 - F-box interaction is structurally conserved among eukaryotes^33^.

Despite the significance of the Sgt1-Skp1 interaction as a key node in the Hsp90 chaperone network, and its association with both Hsp90-dependent activation and SCF-degradation pathways, the molecular details of this interaction are unknown. To gain further insight into this we have determined the 2.8 Å crystal structure of a complex of the interacting domains of yeast Sgt1 and Skp1. Using biophysical and biochemical experiments we show that the crystallographic interaction between individual interacting domains is representative of the interaction between the full-length proteins in solution. Yeast genetic experiments show that the TPR-mediated interaction of Sgt1 with Skp1 contributes an essential function of the cochaperone, and that, by contrast, Sgt1 dimerization mediated by the TPR-interface identified *in vitro* is not required for yeast viability.

## Results

The Sgt1 TPR domain is sufficient for the interaction with Skp1. Previous studies^19^,^20^ have shown that the interaction between Sgt1 and Skp1 is primarily mediated by Sgt1’s TPR domain, with some studies suggesting that Hsp90 may be required for this interaction^19^. The TPR domain is also the site of *S. cerevisiae* Sgt1 dimerization *in vivo* with a reported dimerization K_d_ of 20 nM^21^,^22^.

To confirm that our recombinant full-length Sgt1 is dimeric we carried out sedimentation velocity-analytical ultracentrifugation (SV-AUC) from 4.5 to 22.3 μM Sgt1 (corresponding to 0.2 to 1.0 mg/mL). The AUC data were fit to a continuous c(s) distribution model with a single f/fo (Fig. 2A). At these concentrations the main species (96–98%) had an average corrected sedimentation coefficient of 4.0 S with a molecular weight corresponding to a dimer (theoretical dimer weight 89720 Da) (Supplementary Table S1). A small concentration-dependent change in sedimentation coefficient and estimated molar mass was observed which may be attributable to an oligomerisation or dissociation event in the timescale of the experiment. We therefore used native nano-electrospray mass spectrometry (nESI-MS) at identical concentrations to the SV-AUC experiments to further report on the oligomeric state of Sgt1. The spectra were deconvoluted with Amphitrite^34^ (Fig. 2B) to quantify the contribution of different oligomeric species to the total spectra (Supplementary Table S2). A non-equilibrium, concentration-dependent change in the oligomerisation was detected in the gas phase. At these concentrations, dimeric Sgt1 is the predominant species (48–69%) with subpopulations of both monomer and trimer. Together these results suggest that at experimental working concentrations *S. cerevisiae* Sgt1 is dimeric in solution with a minor population of both monomer and trimer.

To investigate the interaction of Sgt1 with Skp1 using purified proteins *in vitro*, we compared the affinity of Skp1 for full-length Sgt1 and an Sgt1 TPR domain construct (Sgt1TPR, comprising residues 1–150) using isothermal titration calorimetry (ITC). Data were fit using a single site model assuming dimeric Sgt1. For both full-length Sgt1 and Sgt1TPR a stoichiometry of one Sgt1 dimer to one Skp1 monomer was obtained, with both interactions having a K_d_ of 0.6–0.7 μM (Fig. 2C,D), confirming that the Sgt1 TPR domain is sufficient for the Skp1 interaction *in vitro*. Nonetheless the interactions have distinct thermodynamic signatures suggesting a more complex interaction for the full-length proteins.

### Skp1-Sgt1 crystal structure

**Figure 1:**
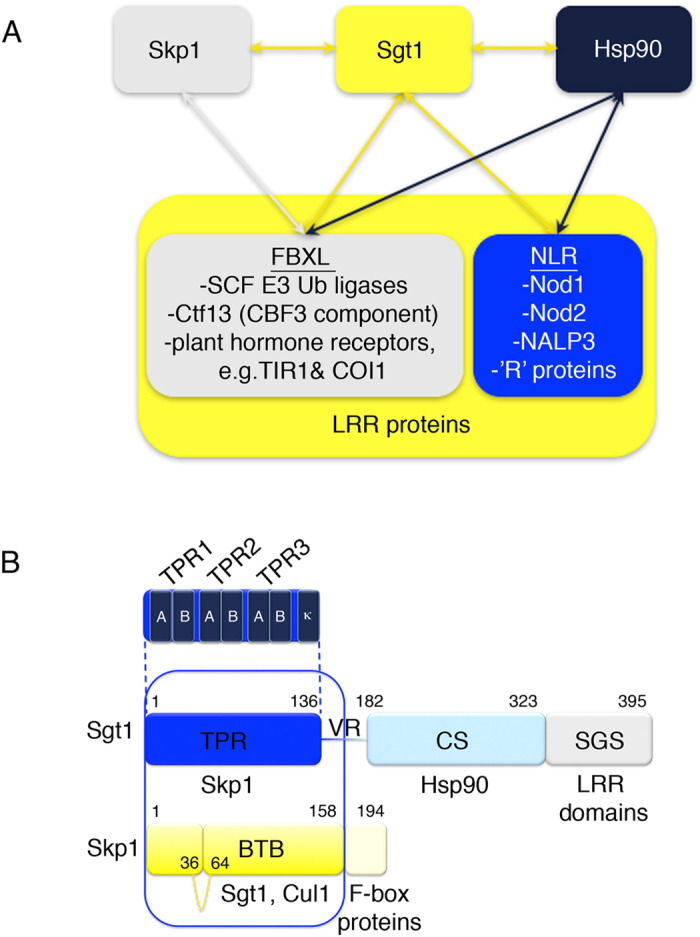
Interactions and Domain Structure of Sgt1 and Skp1. (**A**) Sgt1 recruits Hsp90 function to Leucine Rich Repeat (LRR)-containing proteins. A subset of LRR proteins contain an F-box motif (FBXL proteins) and in these cases Skp1 also interacts with Sgt1. (**B**) Domain structure of Sgt1 and Skp1 with each domain labeled by its structural fold. The individual helices of the TPR motifs within the Sgt1 TPR domain are labeled. The crystallised complex is highlighted in a blue box. An unconserved loop in *S. cerevisiae* Skp1 from residues 36–64 is shown as a dashed yellow line. Known interaction partners are listed below the domain with which they associate.

To investigate the nature of Sgt1 dimerization and its interaction with Skp1 we carried out crystallization screens for the complex between Sgt1TPR and Skp1. Crystals were obtained that diffracted to 2.8 Å (Supplementary Table S3) of a complex comprising Sgt1TPR and a truncated construct of Skp1, Skp1BTB∆. The Skp1BTB∆ construct comprises the N-terminal BTB/POZ domain in which an unconserved loop that is predicted to be unstructured (residues 36–64, Fig. 1B) is replaced by an alanine-serine linker.

The asymmetric unit contains 3 copies of Sgt1TPR (chains A, B & C) and a single copy of Skp1BTB∆ (Fig. 3A). Chains B & C of Sgt1TPR have continuous electron density from Pro2 to Asn136. Chain A is less well-defined with some missing density in loop regions. The Skp1 density is continuous except for residues 65–74, which are C-terminal to the Ala-Ser linker described above, and a loop connecting helices 3 & 4.

The Sgt1 TPR domain comprises 3 TPR motifs, each of two antiparallel helices (A & B), and a solvating helix (κ). These pack together in a right-handed superhelix in which the A & B helices form concave and convex faces respectively (Fig. 3A). The average RMSD of Cα atoms for the individual subunits within the asymmetric unit is 0.49 Å. Structural alignment of Sgt1TPR with TPR domains with high sequence identity to Sgt1 (PPP5, Sgt, CTPR3 & Hop TPR1 with PDBs 1A17, 2VYI, 1NAO & 1ELW respectively) reveals that helices 3 A & 2B are extended compared to canonical TPR motifs, by one and two helical turns respectively. In addition there is an insertion of 8 amino acids between helices 2 A & 2B that includes the turn of a 3_10_ helix. Sequence alignment of Sgt1 homologs suggests that these differences are specific to ascomycete fungi and are absent from metazoans and plants (Supplementary Figure S1).

The overall structure of the BTB/POZ domain of Skp1 in this complex is largely unchanged compared with previously determined structures (RMSD = 0.914 Å)^33^,^35^. It comprises 2 subdomains: an N-terminal subdomain of a 3-stranded β-sheet packed on top of helices α1 and α2, and a C-terminal subdomain of 2 pairs of anti-parallel helices packed perpendicular to each other.

Analysis of the interfaces in the crystal using the EPPIC server^36^, which uses a combination of conservation and geometry-based metrics to distinguish crystallographic from biological interfaces, suggest that the interfaces within the asymmetric unit are likely to be biologically relevant. Composite omit maps for these interfaces are shown in Supplementary Figure S2.

### Skp1 binds in the concave face of the Sgt1 TPR domain

A single copy of Sgt1TPR contributes the entire interaction interface with the N-terminal subdomain of Skp1BTB∆. Skp1 sits within the upper half of the concave face generated by the Sgt1 TPR motif repeats (Fig. 3A). Residues from the α2-helix and β3-strand of Skp1BTB∆ form the interface with residues from Sgt1 TPR1A, 2 A, 3 A and the capping helix.

The Sgt1TPR-Skp1BTB∆ interface in the asymmetric unit buries 597.6 Å^2^. Although relatively small for a protein-protein interface, all charged, polar and hydrophobic Skp1 interfacial residues are either completely or strongly conserved throughout fungi, metazoans and plants with the exception of Leu28, which is a threonine in all other species. Three central residues at the Sgt1 interface are strongly conserved: Arg93 is strictly conserved, Trp127 is strongly conserved (Trp in metazoans and Leu in plants), and Tyr15, which interacts with Skp1 via its benzyl group, is Leu in most metazoans and Phe in plants (Supplementary Figure S1).

In this interface the α2-helix of Skp1BTB∆is amphipathic and forms a bipartite interface with Sgt1 in which half is hydrophobic, and half is electrostatic (Fig. 3B). Residues Lys30 and Asp35 of Skp1 contribute salt bridges with Asp16 and Arg93 of Sgt1 (Supplementary Figure S2). These electrostatic interactions are supported by additional hydrogen bonds between Tyr11, Tyr15, Lys50, Asn100 of Sgt1 and Asn34 and Asp35 of Skp1. The hydrophobic core is formed by the other side of the amphipathic α2-helix, with Leu28, Leu29 and Tyr32 of Skp1 interacting with Thr123, Leu126 and Trp127 on the capping helix of Sgt1. Met77 and Pro78 on the β3-strand of Skp1BTB∆ also contribute to this interface.

To test our observations in the context of the full-length proteins in solution we mutated Sgt1 residues at the Sgt1-Skp1 interface and investigated the ability of these His_6_-tagged Sgt1 mutants to interact with untagged wild-type Skp1 in a pulldown assay (Fig. 3C). Mutation of highly conserved Arg93 and Trp127 to Ala results in complete loss of binding of the full-length proteins in solution, whereas mutation of Leu126, a poorly conserved residue contributing a van der Waals interaction at the edge of the interface, to Ala, does not significantly affect binding compared to the WT. These results are consistent with the crystallographic interface providing the primary interaction between full-length Sgt1 and Skp1 in solution.

### A conserved charged sequence is essential for the Sgt1-Skp1 interaction

In *S. cerevisiae* an unconserved low sequence complexity region (LCR), comprising residues 36–64 is followed by an acidic stretch of aspartate and glutamate residues that is also partially conserved in metazoans (residues 69–74) (Fig. 4A). The LCR does not contribute essential function since human Skp1, in which the LCR is absent, can rescue the temperature-sensitive growth of the skp1–12 mutant^37^. Complexes with Skp1 including the LCR were refractory to crystallization, probably as a consequence of its predicted disorder. This has also been observed in previously determined Skp1-F-box crystal structures^32^,^33^. In our Sgt1TPR-Skp1BTB∆ structure the LCR is replaced by an alanine-serine linker and electron density for the alanine is visible at the edge of the Sgt1-Skp1 interface.

Electron density was missing for residues 65–74, immediately adjacent to the truncated LCR, despite being present in the crystallised construct. Since the disorder of this conserved segment in the crystal structure may be a consequence of the LCR truncation, possibly by restricting the conformational space available, we tested if these residues may contribute to the interaction with Sgt1 in the context of the full-length proteins. We generated two mutants of Skp1, referred to as Skp1∆1 and Skp1∆2 (Fig. 4A). In both mutants the LCR residues are retained but in Skp1∆1 residues 65–74 are deleted, whereas in Skp1∆2 they are replaced by a series of alanine and lysine residues that introduce a charge reversal of equivalent length. ITC of these mutants, at concentrations equal to those used to assess the affinity of the Sgt1- Skp1 WT complex, shows that Skp1∆1 has a weaker interaction with Sgt1 than the wild-type protein (Fig. 4B) although since the integrated curve has poorly defined plateaus, the estimated K_d_ for this mutant is limited by the quality of the fit. By contrast no binding isotherm could be detected for Skp1∆2 (Fig. 4C). These results suggest that, although disordered in the crystal, the conserved charged sequence from residues 65–74 does contribute positively to the interaction between Sgt1 and Skp1. The surface of Sgt1 in the vicinity of this missing region (near Val76) may provide a binding surface for some of these acidic residues (Fig. 4D) as it is predominantly basic, and several of the residues contributing to this patch (Lys102 & Arg 130) are conserved in higher eukaryotes.

### Yeast specific insertions in the Sgt1 TPR domain are responsible for *in vitro* oligomerization

Dimerization of Sgt1 has been reported to play a role in the assembly of the yeast kinetochore, with temperature-sensitive mutants that disrupt dimerization *in vivo* having an increased chromosome missegregation phenotype^22^. This may be a consequence of impaired formation of the kinetochore complex CBF3^27^. We therefore investigated if this dimerization interface was represented in our crystal structure.

The three copies of Sgt1TPR in the asymmetric unit form two identical dimeric interfaces (between C-A and A-B) with an rmsd of 1.46 Å in an all-atom superposition (Supplementary Figure S1). The average interface area is 601.5 Å^2^ (C-A = 596.5 Å^2^; A-B = 606.5 Å^2^) and occurs via a perpendicular arrangement of subunits in which the convex face of one protomer packs against the intra-TPR loops of another. The yeast specific insertion between TPR2A and 2B of both protomers contributes the majority of the interactions of this interface (Supplementary Figure S1). At its core His59 makes both a salt-bridge with Asp57 and a cation-π interaction with Trp58 on the other protomer (Fig. 5A). This is supported by additional van der Waals interactions between Thr63 and Phe53. These residues are highly conserved amongst ascomycetes (Supplementary Figure S1). The periphery of the interface comprises a number of H-bond interactions including from Asp57, Ser60 and Asp61, all located in the loop insertion, bonding to His59, Asn100 and Arg130 respectively (Fig. 5A).

In order to test whether the interface is relevant in full-length Sgt1 the mutations His59 to Ala and Asp61 to Arg were introduced into an N-terminal His_6_-tagged full-length Sgt1 and their effects on dimerization were assessed. In size exclusion chromatography (SEC), Sgt1-H59A has a larger elution volume than WT, indicative of a smaller oligomeric state, whereas Sgt1-D61R has an elution volume intermediate between the two (Fig. 5B). These qualitative results are consistent with the contribution of each residue to the interface: His59 contributes two interactions at the centre of the interface, and its mutation disrupts dimerization of Sgt1; Asp61 forms a peripheral salt-bridge and mutation of this residue causes a mixture of higher and lower oligomers that interconvert on the timescale of the experiment.

Since all full-length Sgt1 constructs elute at a larger volume than expected for their molecular weight, probably due to an elongated structure, quantitative results were obtained from SV-AUC and nESI-MS experiments. The corrected sedimentation coefficient of 4.4 S for His_6_-Sgt1 WT reflects a slight increase in the c(s) compared to the untagged WT protein, as a consequence of the N-terminal His_6_ tag. The H59A mutant has a corrected sedimentation coefficient of 2.5 S, with an estimated molecular weight 41 kDa indicating a monomeric species (Fig. 5C, Supplementary Table S1). nESI-MS also shows that the H59A mutation significantly disrupts the oligomerization and that this mutant of Sgt1 is predominantly monomeric (Fig. 5D, Supplementary Table S2). By contrast, the D61R mutant retains some wild-type characteristics with a major population of dimer, but with a loss of trimer relative to wild-type. Therefore the solution and nESI-MS data support the crystallographic interface as contributing to the oligomerisation of full-length Sgt1 *in vitro*.

The presence of a small population of trimers of full-length Sgt1 as determined by nESI-MS and the observation of 3 copies of the TPR domain in the asymmetric unit suggests that this oligomerisation state is partially accessible to the full-length protein but is restricted due to steric crowding from the CS and SGS domains. To investigate this possibility, the oligomeric properties of the Sgt1TPR construct were tested using SV-AUC, nESI-MS and SEC-MALS (Supplementary Figure S3). All three techniques highlight a propensity for this construct to form concentration-dependent trimers, supporting the conclusion that the artificial truncation of the CS and SGS domains relieves steric crowding allowing trimer formation in the TPR domain construct.

### Skp1 interaction is a requirement for the essential Sgt1 function in yeast

Given the significance of the interaction between Sgt1 and Skp1 for the SCF pathway and for assembly of the yeast kinetochore^1^,^38^ we sought to validate our crystallographic and biophysical data *in vivo*. Wild type Sgt1-His_6_ expressed under the Sgt1 native promoter as the sole Sgt1 in yeast provided functionality, and the strain was not temperature-sensitive. The mutations R93A and W127A at the Sgt1TPR-Skp1 interface that prevented the interaction of the full-length proteins *in vitro* were not viable, as seen by absence of growth at restrictive (37 °C) and non-restrictive (28 °C) temperatures (Fig. 6A). However the mutations H59A and D61R that completely or partially disrupted oligomerization of Sgt1 *in vitro* were both found to provide viability and were not temperature-sensitive. Wild type and mutant Sgt1 proteins expressed under the Sgt1 promoter were expressed to comparable levels (Fig. 6B). These *in vivo* results support our *in vitro* analysis, confirming the residues shown to be important for the interaction between Sgt1 and Skp1 in our crystal structure are vital for the Sgt1-Skp1 interaction *in vivo* and that this interaction is essential for Sgt1 function.

## Discussion

Sgt1 is an essential cochaperone of Hsp90 yet it is poorly characterized at both structural and mechanistic levels. We present the crystal structure of the TPR domain of Sgt1 with Skp1, an interaction partner that connects Sgt1 to kinetochore assembly and SCF activity in *S. cerevisiae*^1^.

**Figure 2:**
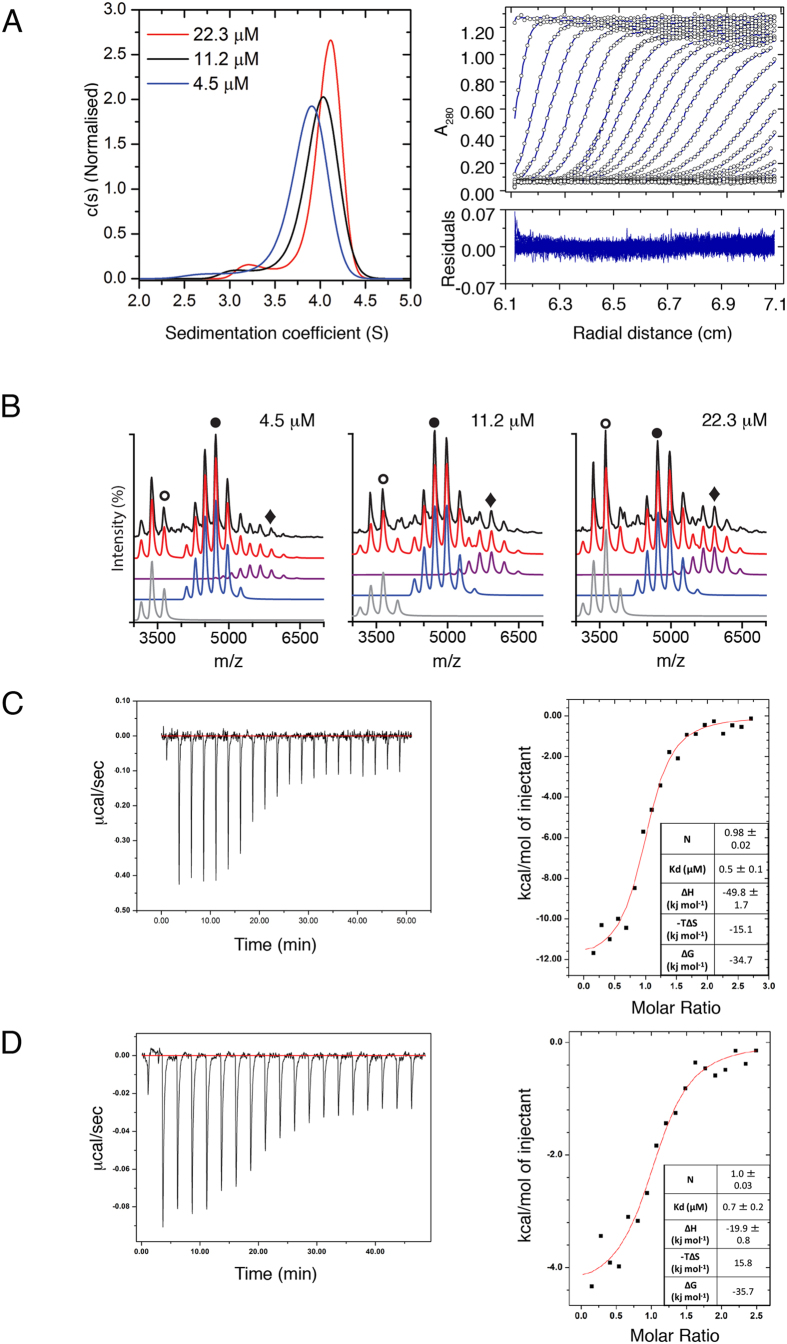
Biophysical Characterisation of Sgt1 and Its Interaction with Skp1. **(A)** c(s) distributions for Sgt1 FL at 4.5 μM, 11.2 μM & 22.3 μM monomer and corresponding boundary fits and residuals for the sample at 4.5 μM generated by SV-AUC. Supplementary Table S1 details the sedimentation coefficients, MW and f/fo. **(B)** Native nESI-MS spectra of full-length Sgt1 at 4.5 μM, 11.2 μM and 22.3 μM. Charge state series corresponding to monomer, dimer, and trimer are indicated in grey, blue and purple respectively. Charge states: wt + 13 ○, (wt)_2_ + 20 ●, (wt)_3_ + 24 ♦. Spectra were deconvoluted to individual charge state series using Amphitrite^34^ with the raw data shown in black and the sum of simulated spectra in red. Supplementary Table S2 details the percentage contribution of individual species at each concentration. **(C)** Left: Raw isothermal titration calorimetry (ITC) data for the interaction between full-length Sgt1 (12 μM dimer) and full-length Skp1 (160 μM) at 25 °C. Right: Dilution-corrected and integrated heats for the ITC data are fitted according to a single binding site model assuming dimeric Sgt1. **(D)** As Figure C but for the interaction between Sgt1TPR (10 μM dimer) and full-length Skp1 (140 μM) at 25 °C.

**Figure 3:**
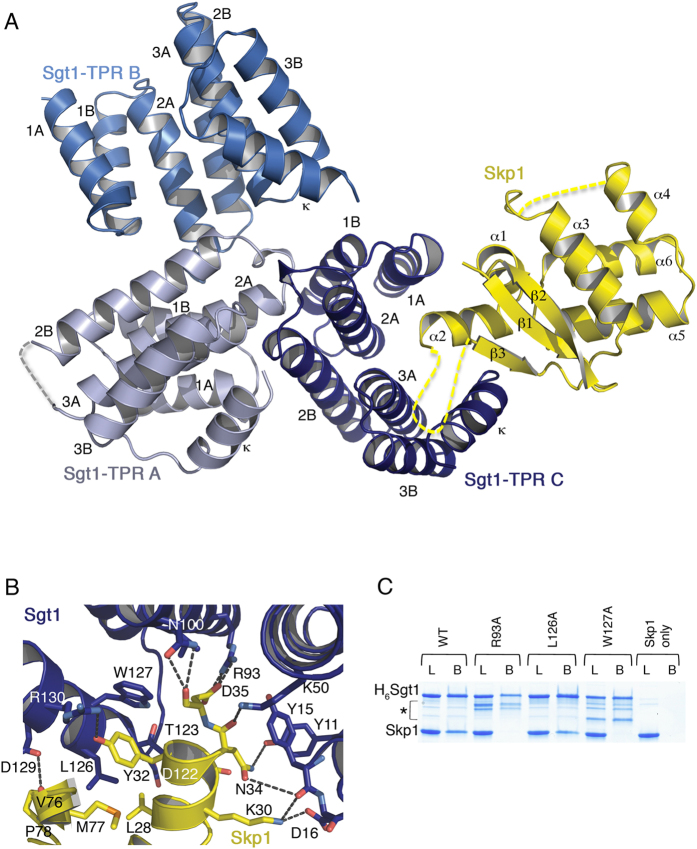
Crystal Structure of The Skp1 TPR Domain in Complex With Skp1. **(A)** The asymmetric unit of the complex contains 3 subunits of Sgt1TPR (different shades of blue) and one Skp1BTB∆ (yellow). The individual TPR motifs are labeled within each TPR domain. Loops that are not visible in the crystal structure are shown as dashed lines. **(B)** The Sgt1TPR-Skp1BTB∆ interface. Residues that make the closest van der Waals contacts and hydrogen bonds are shown. Domains coloured as (**A**). **(C)** Pulldown of full-length Skp1 using His_6_-tagged wild type Sgt1 (WT) and indicated mutants on Ni resin. L = 10% load, B = bound. * = Degradation products of Sgt1 that copurify with the full-length protein. The full-length gel is presented in Supplementary Figure S6A.

Our ITC experiments show that a single Skp1 interacts with the TPR domain of the Sgt1 dimer (Fig. 2C,D), and our crystal structure indicates that only one TPR domain within the Sgt1 dimer contributes to this interaction (Fig. 3A). A pivotal interaction in this interface is a salt-bridge between Sgt1-Arg93 and Skp1-Asp35, and our structure-guided mutagenesis experiments using full-length proteins show that mutation of Arg93 alone is sufficient to prevent Skp1 binding *in vitro* (Fig. 3C) and cause loss of viability *in vivo* (Fig. 6). Together these data support the conclusion that the primary Sgt1-Skp1 interface utilizes the concave groove of the TPR domain identified in the crystal structure.

The interaction of Skp1 with full-length Sgt1 has a smaller exothermic component (Fig. 2C,D) than the interaction with Sgt1TPR, indicative of enthalpic contributions from residues outside of the TPR domain of Sgt1. These could arise from either additional Skp1 contacts outside the TPR domain, or conformational changes in full-length Sgt1 on association with Skp1 that are not recapitulated in the interaction of the TPR domain alone. Immunoprecipitation studies in reticulocyte lysates of *in vitro* translated Sgt1 truncations suggest that both possibilities could occur^19^, however since the consequences of the R93A mutation in Sgt1 are so striking in all our experiments, the latter is the more likely scenario. A detailed understanding of any such conformational changes will require structural information for full-length Sgt1.

Many Hsp90 cochaperones contain TPR domains that interact with Hsp90’s C-terminal MEEVD motif^39^,^40^,^41^,^42^,^43^. Central to this interaction is the “carboxylate clamp”, in which the carboxylates of the C-terminus and the sidechain of the final aspartate are anchored by basic and polar residues from each repeat within the concave face of the TPR domain. Specifically, a lysine and an asparagine from TPR1A, an asparagine from TPR2A and a lysine from TPR3A are typically conserved in TPR cochaperones. A structure-based sequence alignment of the Sgt1 TPR domain with MEEVD-interacting cochaperones (Supplementary Figure S4) shows that three out of these four conserved residues are not found in Sgt1, explaining the lack of interaction with the Hsp90 MEEVD-motif. Instead the CS domain of Sgt1 interacts with the N-terminal domain of Hsp90^24^. The fourth residue, Lys8 on TPR1A of Sgt1, is conserved but is remote from the Skp1 interface.

In addition, in many MEEVD-TPR interfaces a further two electrostatic contributions are made: from a lysine on TPR2A and an arginine of TPR3A to the first glutamate and the backbone of the MEEVD peptide respectively. Both of these residues (Lys50 and Arg93) are conserved in Sgt1 and contribute to the Skp1 interface (Fig. 3B). Arg93 plays a critical role as its mutation results in the complete loss of interaction with Skp1 in pulldown assays (Fig. 3C) and, most significantly, the loss of viability *in vivo* (Fig. 6).

**Figure 4:**
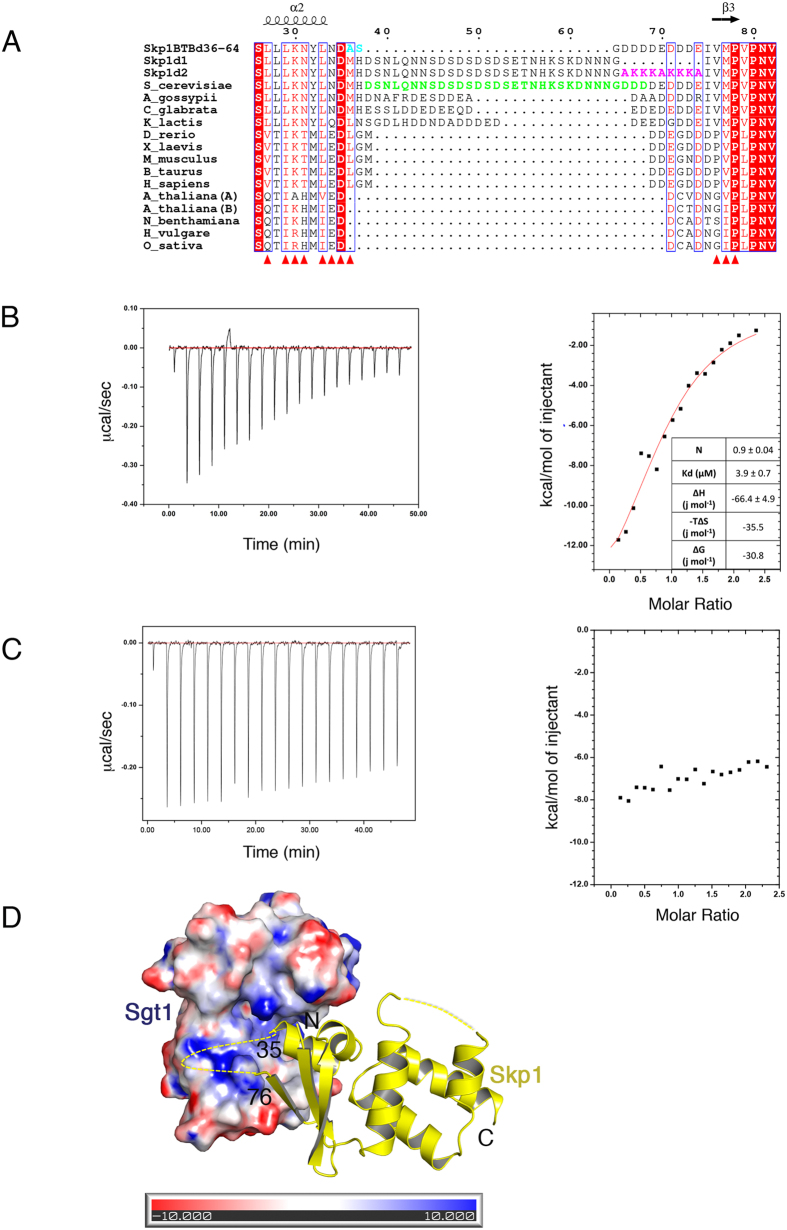
Contribution of a Charged Sequence to Sgt1-Skp1 Association. **(A)** Sequence alignment of Skp1 homologs including the Skp1BTB∆ (Skp1BTBd36–64) construct used for crystallization, Skp1∆1 (Skp1d1) and Skp1∆2 (Skp1d2) mutants. *S. cerevisiae* Skp1 has a loop (green) that is not conserved in higher eukaryotes. The loop is replaced by an Ala-Ser linker (cyan) in Skp1BTB∆. The mutated residues in Skp1∆2 are coloured magenta. White text on a red background indicates strict identity, red text indicates similarity in a group, a blue frame indicates similarity across groups. Residues that contribute to the interface between Sgt1 and Skp1 are shown as red triangles. Generated using ESPript^56^. **(B)** Left: Raw ITC data for the interaction between Sgt1 (13 μM dimer) and Skp1Δ1 (130 μM) at 25 °C. Right: Dilution-corrected and integrated heats for the ITC data are fitted according to a single binding site model assuming dimeric Sgt1. **(C)** As Figure B but for the interaction between Sgt1 (12 μM dimer) and Skp1∆2 (145 μM) at 25 °C. **(D)** The complex of Sgt1TPR (electrostatic surface potential ± 10 kT/e), generated using PDB2PQR and APBS^57^,^58^ and Skp1BTB∆ (shown in cartoon). The unconserved deleted loop (residues 36–64) and following acidic stretch (residues 65–73) are shown as a dotted line.

**Figure 5:**
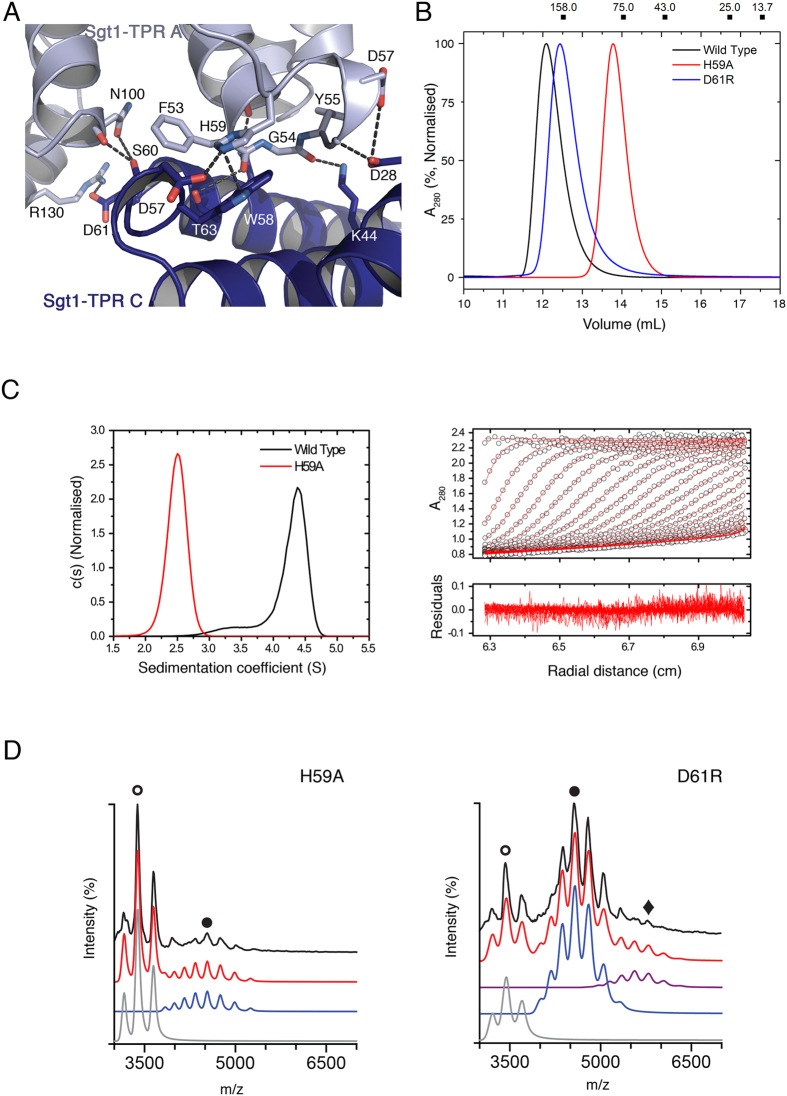
The Sgt1 Dimerization Interface. **(A)** A detailed view of the oligomerization interface of Sgt1TPR. Residues that make the closest van der Waals contacts and hydrogen bonds are shown. **(B)** Size exclusion chromatography of full-length wild-type Sgt1 and the mutants H59A and D61R at 22.3 μM. Molecular weight standards for the column are shown above the chromatograph. **(C)** The c(s) distribution for full-length His_6_-tagged WT and H59A Sgt1 at 11.2 μM in SV-AUC and corresponding boundary fits and residuals for the H59A mutant. (**D**) Native nESI-MS spectra of the full-length Sgt1 H59A and D61R mutants. Charge states: monomer + 14 ○; dimer + 20 ●; trimer + 24 ♦. Data were deconvoluted with Amphitrite^34^ and are represented directly below each spectrum. The raw data are shown in black and the sum of simulated spectra in red. Charge state series corresponding to monomer, dimer, and trimer are indicated in grey, blue and purple respectively. Supplementary Table S2 details the percentage contribution of individual species at each concentration.

The truncated, non-essential, unconserved LCR (residues 36–64) and a partially conserved stretch of acidic polypeptide (residues 66–74) are positioned at the edge of the interface in the crystal structure. These regions of Skp1 may enlarge the interface between the full-length proteins and our ITC analysis (Fig. 4) supports this conclusion, since both deletion and mutation of the acidic residues negatively affects the dissociation constant of the full-length proteins. In addition, several crystal structures of MEEVD-interacting TPR cochaperones^39^,^40^,^41^,^42^,^43^, the methionine of the MEEVD motif binds in a shallow hydrophobic pocket on the concave face of the TPR. The proximity of the truncated methionine, Met36, to the highly conserved Sgt1-Trp127, suggests that a similar mechanism could contribute to the interface in the full-length complex.

Mapping the location of mutations that give rise to temperature-sensitive (ts) phenotypes to the domain structure of Sgt1 shows that those within the TPR domain cause a G2/M arrest phenotype associated with impaired kinetochore formation^1^,^19^. The structure presented here of the interacting domains of Sgt1 and Skp1 provides a structural basis for understanding these *in vivo* observations.

**Figure 6:**
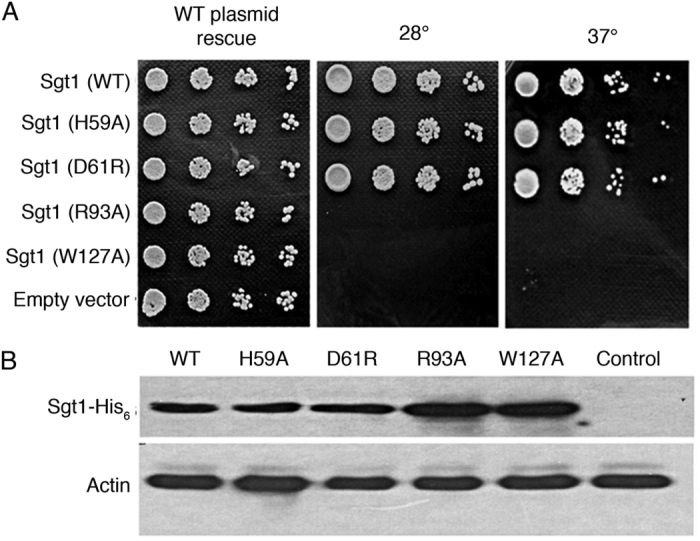
Yeast genetic analysis. (**A**) Growth of *S. cerevisiae* JJ345 cells expressing wild-type (WT) Sgt1 or indicated mutants. Overnight 28 °C SD-Trp cultures were serially diluted, then pinned onto SD-Trp agar and photographed after 2 days of growth at 28 °C and 37 °C. (**B**) Analysis of Sgt1 level in 28 °C SD-Trp cultures expressing WT Sgt1 and either WT or mutant Sgt1-His_6_. Actin was measured as a loading control. Full-length blots are presented in Supplementary Figure S6B.

All G2/M mutant alleles result in either the replacement of a leucine with a proline in a helix, which will result in helix unfolding (sgt1–3, sgt1–6, sgt1–7) (e.g. Supplementary Figure S1), or the introduction of a polar residue in a hydrophobic core (sgt1–12). Such structurally disruptive mutations are likely to destabilize the domain, and as such cause partial loss of both dimerization and Skp1 interaction. Indeed all TPR domain ts-mutants reportedly lose Skp1 interaction in a yeast two-hybrid screen^19^. Our data suggest that the G2/M phenotype of Sgt1 ts-mutants is a consequence of disruption of Skp1 association with Sgt1, rather than its self-association, as disruption of the dimerization interface that we identified *in vitro* does not result in temperature sensitivity (Fig. 6). In addition, our monomeric Sgt1 H59A mutant can pull-down Skp1 as efficiently as WT in an *in vitro* assay using purified proteins (Supplementary Figure S5).

However since derivatives of G2/M mutant alleles can be rescued by attaching an artificial dimerization domain^22^, and as phosphorylation of the C-terminal SGS domain of yeast Sgt1 negatively regulates both dimerization and the association with Skp1 *in vivo*^27^ (despite the modification being remote from the dimerization interface identified in our crystal structure) we cannot exclude the possibility that additional or different dimerization interfaces exist *in vivo*.

G2/M mutant Sgt1 alleles display benomyl sensitivity, chromosome missegregation and loss of CEN DNA binding by the CBF3 complex of the yeast kinetochore^1^.Within CBF3, Skp1 forms a heterodimer with the FBXL Ctf13^4^ which is an Hsp90 client protein^19^,^44^,^45^ comprising an N-terminal F-box and C-terminal LRR domain. Superposition of the Skp1-Skp2 complex^32^, the closest homologous complex of known structure, on the Sgt1TPR-Skp1BTB∆ crystal structure shows that the interactions of Skp1 with both Sgt1 and Skp2 are structurally compatible (Supplementary Figure S4), as demonstrated biochemically^24^. The Sgt1-Skp1 complex can therefore form a direct bridge between FBXL proteins, such as Ctf13, and Hsp90.

The Sgt1-Skp1 complex is also implicated in the SCF pathway in *S. cerevisiae*. In the active SCF E3 ubiquitin ligase, Skp1 interacts both with a substrate-recruiting component and Cul1, which is a structural scaffold for the E3 ligase component of the enzyme. The superposition of the Sgt1TPR-Skp1BTB∆ structure with the Skp1-Cul1 complex^31^ (Supplementary Figure S4) reveals that both Cul1 and Sgt1 share a common interaction surface on Skp1, and that they would therefore compete for Skp1 binding. In humans, Cul1 will displace Sgt1 from Skp1, as the Cul1-Skp1 interaction is 2 orders of magnitude tighter than the Sgt1-Skp1 interaction^24^. Since unlicensed SCF activity would be detrimental to the cell, specificity of substrate binding to SCF complexes is tightly controlled by a range of mechanisms, including phosphorylation of the substrate and interaction with adaptor molecules^46^. The Sgt1-Skp1 interaction may therefore serve to provide an additional layer of regulation.

In humans and plants Sgt1’s function in the SCF E3 ligase pathway is conserved, and both Sgt1 and Skp1 have been also shown to be required for efficient kinetochore formation in humans^47^. The Sgt1TPR-Skp1BTB∆ crystal structure presented here provides an additional piece of the structural jigsaw that links Hsp90 to these pathways. However the function of Sgt1 in each system may be context dependent and will require a molecular understanding of how Sgt1 interacts with client proteins and mechanistic details of Hsp90 function in these pathways.

## Methods

### Protein Expression and Purification

Untagged full-length Skp1 from *S. cerevisiae* in pET22b was a gift from Martin Singleton. Untagged Skp1BTB∆ (residues 1–158) was subcloned using *Nde*I and *BamH*I sites into pRsetA with a deletion of residues 34–64 and replaced by Ala-Ser. We have used full-length numbering of Skp1 for residues C-terminal to the loop deletion. Expression of the full-length Skp1 and Skp1BTB∆ was performed in BL21 Star (DE3) (Invitrogen) and T7 Express LysY/I^q^ (NEB) respectively, supplemented with the pRARE (Novagen) plasmid.

Untagged full-length Sgt1 and Sgt1TPR (residues 1–150) from *S. cerevisiae* were cloned in MCSII of pColA-Duet (Novagen) using *Nde*I and *BamH*I. His-tagged full-length Sgt1 and mutants thereof were cloned into pET28a (Novagen) using *Nde*I and *Xho*I. Untagged full-length Sgt1 was expressed in BL21 Star (DE3); all other constructs were expressed using T7 Express LysY/I^q^.

All protein expression was induced with 1 mM IPTG and was continued overnight at 18 °C. Skp1 constructs were purified by 2 rounds of anion exchange (HiTrap Q HP, GE Healthcare) followed by gel filtration (Superdex 75, GE Healthcare) in 50 mM Tris pH 7.5, 2 mM DTT, 1 mM EDTA, 150 mM NaCl. Untagged Sgt1 constructs were purified using 2 rounds of cation exchange (HiTrap SP FF, GE Healthcare) followed by gel filtration (either Superdex 200 or Superdex 75 depending on construct molecular weight) in 25 mM HEPES pH7.0, 150 mM NaCl, 2 mM DTT, 1 mM EDTA. For the purification of His-tagged Sgt1 constructs cation exchange was replaced with a single HisTrap FF (GE Healthcare) column in a buffer of 50 mM Tris pH7.5, 300 mM NaCl, 10% glycerol. The remaining steps were as for the Sgt1 untagged constructs.

### Isothermal Titration Calorimetry

A VP-ITC Microcalorimeter (MicroCal) was used for ITC experiments. All proteins were buffer exchanged into 20 mM HEPES pH7.0, 150 mM NaCl, 1 mM β-mercaptoethanol. Typically 20 injections of 130–160 μM Skp1 were injected into 6–13 μM Sgt1 dimer at 25 °C. Heats of dilution were subtracted and data fitted assuming a single site per Sgt1 dimer allowing three floating variables (stoichiometry, binding constant and enthalpy). Heats of dilution were measured by injecting Skp1 into buffer. Data was corrected for Skp1 dilution into buffer alone, and integrated using Origin software. The integrated heats were fitted using nonlinear regression using a single site model.

### Crystallisation, data collection and structure determination

Purified Sgt1TPR and Skp1BTB∆ were mixed in a 2:1 molar ratio and concentrated to 32 mg/mL total protein and crystallised using vapour diffusion. Rod shaped crystals were obtained in 0.325 M MgCl_2_, 22.5% PEG-6000, 0.1 M Tris-HCl pH 8 at 4 °C. Crystals were frozen using 20% ethylene glycol as a cryoprotectant. Data were collected to 2.8 Å at IO4, Diamond Light Source, UK. Data were processed using XDS^48^,^49^. Molecular replacement attempts using either an ensemble of homologous TPR domains or Skp1 failed, therefore crystals were grown with SeMet-Sgt1TPR and unlabelled Skp1BTB∆ and a SAD dataset was collected. Initial phases were obtained from the AUTO-Rickshaw server^49^,^50^, which placed 6 selenium atoms using SHELXD in the spacegroup P3_2_21. 88.9% of the TPR scattering mass in the asymmetric unit was built into electron density automatically using HELICAP and BUCCANEER. Molecular replacement using Phaser^51^ was then used to place the Skp1BTB∆ domain. The remaining model was built in COOT^52^ and refined in Buster (Bricogne *et al*., 2011) using automatic NCS restraints^53^ and TLS parameters. Data collection and refinement parameters are listed in Supplementary Table S3. Simulated annealing omit maps at key interfacial residues were calculated in Phenix and are shown in Supplementary Figure S2.

### Mass Spectrometry

Prior to mass spectrometry analysis, samples were buffer exchanged into 150 mM ammonium acetate pH 7, using Micro Bio-Spin 6 columns (BioRad, Herts. UK). Mass spectrometry experiments were carried out on a first generation Synapt HDMS (Waters Corp., Manchester, UK) mass spectrometer^54^. Instrumental parameters were as follows: source temperature 40 °C, capillary voltage was optimized between 0.9–1.2 kV, cone voltage 80 V, TriWave trap and transfer voltages were kept at a constant 5 and 10 V respectively. Data acquisition and processing were carried out using MassLynx 4.1 software (Waters Corp., Milford, MA, USA). Deconvolution of oligomerization states was performed using Amphitrite^34^.

### *In vitro* protein pulldown assays

All incubations were carried out in 25 mM HEPES pH 7, 100 mM NaCl. 60 μl of Ni Sepharose HP Resin (GE healthcare) was blocked with 1 mg/mL BSA and then pre-incubated with 9 nmoles purified His-Sgt1 (wild-type or mutant) for 1 hour before addition of 20 nmoles of purified Skp1 for a further hour. Resin was washed three – four times with buffer and the reaction eluted with SDS-PAGE loading buffer.

### Sedimentation Velocity Analytical Ultracentrifugation

SV-AUC was carried out using a ProteomeLab XL-I (Beckman Coulter) analytical ultracentrifuge at 20 °C. Eight-cell (An-50 Ti) or four-cell (An-60 Ti) rotors (Beckman Coulter) with 12-mm cells were used at a velocity of 42000 or 60000 RPM respectively. Absorbance data were collected at 280 nm. Protein partial specific volume, buffer density and viscosity were calculated using SEDNTERP (Laue *et al*., 1992). The Lamm equation was used to fit sedimentation boundaries of 200 scans in SEDFIT (Schuck, 2000) using a c(s) with one discreet component.

### Genetic Analysis

The yeast strain JJ345^55^, maintained with a URA3 plasmid-based Sgt1, was transformed with a TRP1 plasmid comprising C-terminally His_6_ tagged wild-type and mutant Sgt1 under its own promoter and cured of the URA vector with 5-FOA. Protein levels were determined by Western blot using anti-TetraHis antibody and actin used as a loading control. Temperature sensitivity was determined by spotting 5-fold dilutions of cells onto plates and growing at control (28 °C) and heat stress (37 °C) temperatures and imaged after two days.

## Additional Information

**Accession codes:** The structure is deposited with PDB code 5AN3.

**How to cite this article:** Willhoft, O. *et al*. The crystal structure of the Sgt1-Skp1 complex: the link between Hsp90 and both SCF E3 ubiquitin ligases and kinetochores. *Sci. Rep.* **7**, 41626; doi: 10.1038/srep41626 (2017).

**Publisher's note:** Springer Nature remains neutral with regard to jurisdictional claims in published maps and institutional affiliations.

## Supplementary Material

Supplementary Information
