# Supplementary material for: The crystal structure of the Sgt1-Skp1 complex: the link between Hsp90 and both SCF E3 ubiquitin ligases and kinetochores
Source: Sci Rep. 2017 Jan 31;7:41626. doi: 10.1038/srep41626 (PMC5282575; doi:10.1038/srep41626)
Supplement: Supplementary Information [file srep41626-s1.pdf]

THE CRYSTAL STRUCTURE OF THE SGT1-SKP1 COMPLEX: THE LINK BETWEEN HSP90 AND BOTH SCF E3 UBIQUITIN LIGASES AND KINETOCHORES

Oliver Willhoft, Richard Kerr, Dipali Patel, Wenjuan Zhang, Caezar Al-Jassar, Tina Daviter, Stefan H. Millson, Konstantinos Thalassinos, Cara K. Vaughan

SUPPLEMENTARY FIGURE S1: SGT1 TPR DOMAIN ANALYSIS

A

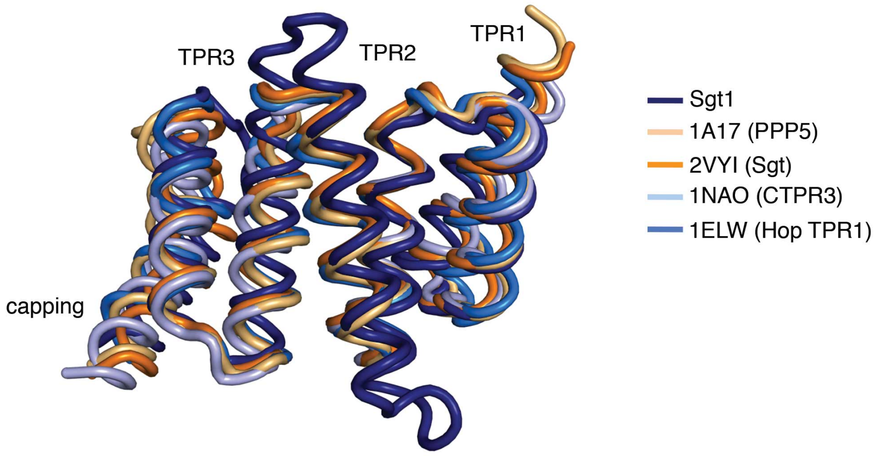

B

```
                                 TPR1A                TPR1B                TPR2A
S_cerevisiae
                 1               10          20          30          40          50
S_cerevisiae     MPVEKD........LKTAYKALYDEKEPLKALHLYDEILKGSPTNLTALIFKAACLEKLY
K_lactis         MPVEKD........LKEAYSLLYDKIEVEKALELYDKVLKQDPNSLNANVYKSVCLEKLY
C_glabrata       MPIEKD........LKVAYDVLYEEGDPKRALELYNRILKESPKALNALIYKAACYEKLY
A_gossypii       MPIEVD........LKNAYRLLYDEKEPEKALELYDNVLQQSSTNLIALIYKAAALEKLY
H_sapiens        MAAAAAGTAT.SQRFFQSFSDALIDEDPQAALEELTKALEQKPDDAQYYCQRAYCHILLG
B_taurus         MAAAAAGPVAAASRLFRSFSDALIEQDPQAALEELTKALEQKPDDAPYYCQRAYCHILLG
M_musculus       MAAAAAGPAS.SQRLFQSFSDALIDGDPQAALEELTKALEQNPDDAQYYCQRAYCHILLG
X_laevis         MTEAGTVPPA......ISFPDSCIGADPHKSLEELTKAIEEKSDCAEYYCQRAYAQILLQ
D_rerio          MATCG...........RYSDSFIDEDPRRALEELNEALGEKADNAEWLCQRAYAYILLK
A_thaliana       MAKE..........LADKAKEAFVDDDFDVAVDLYSKAIDLDPNCAEFFADRAQAYIKLE
H_vulgare        MAAAAASD......LESKAKEAFVDDDFELAAELYTQAIEAGPATAELYADRAQAHIKLG
O_sativa_Jap_    MATAAASD......LESKAKAAFVDDDFELAAELYTQAIEASPATAELYADRAQAHIKLG
N_benthamiana    MASD..........LETRAKEAFIDDHFELAVDLYTQAIAMTPKNAELFADRAQANIKLN

                 insert      TPR2B                       TPR3A               TPR3B
S_cerevisiae
                         60          70          80          90          100         110
S_cerevisiae     FGFSDWHSDATMENAKELLDKALMTAEGRGDRSKIGLVNFRYFVHFFNIKDYELAQSYFK
K_lactis         FRSSSWHNEETLQASYDFLKKALDIAERRGDRSKIGFVYFRFCIHYYNCKKYCLAQEFMQ
C_glabrata       FGDKQYRTVETLDEAKSCLAEAEDVAMERGDRAKIGLVYFRFFVHHFNLKYYKMAETYLK
A_gossypii       YSTKSWHSDGTLEEARGLLEKALDLAEQRGDRGKLALVNFRAFVYHFNRREYAAAEPYMT
H_sapiens        N..........YCVAVADAKKSLEL......NPNNSTAMLRKGICEYHEKNYAAALETFT
B_taurus         N..........YSDAVADAKKSLEL......NPNSSTALLRKGICEYHEKNYAAALETFT
M_musculus       K..........YRDGIADVKKSLEL......NPNNCTALLRKGICEYHEKDYASALETFA
X_laevis         N..........YNDAVLDAKRSLEL......QPNNASAFLRKGEAEFHLQNYSSAEESFR
D_rerio          E..........YTKAIDDAKKAQDL......QPGLALPFLRIGIAEYHLHNFVAAHQALT
A_thaliana       S..........FTEAVADANKAIEL......DPSLTKAYLRKGTACMKLEEYRTAKTALE
H_vulgare        S..........YTEAVADANKAIEL......DPSMHKAYLRKGSACIKLEEYQTAKAALE
O_sativa_Jap_    N..........YTEAVADANKAIEL......DPSMHKAYLRKGAACIRLEEYQTAKAALE
N_benthamiana    Y..........FTEAVVDANKAIEL......DPSMSKAYLRKGLACMKLEEYQTAKAALE

                             capping
S_cerevisiae
                         120         130         140         150
S_cerevisiae     KAKNLGYVDDTLPLWEDRLETKLNKKNKKQKDSTNK....HT
K_lactis         KCESYGFKDDTLPMWKHNIDTAIRKHVKKDVMDERA...KAV
C_glabrata       RAKSYGYADPTLALWEDKLKNKLAKMNSEHGDDTDKKGKSDL
A_gossypii       RAKQLGYDDATLGIWEANLEKKLKKAAAPGAAENRD....EP
H_sapiens        EGQKLDSADANFSVWIKRCQEAQNGSESEV............
B_taurus         EGQKLNSADADLTAWIKRCQEAQNGSQPEV............
M_musculus       EGQKLDSTDTNFDTWIKRCQEIQNGSESEV............
X_laevis         KGQMLDTSTPTFPTWIKRCEEKLNVSAEEQL...........
D_rerio          AGRDLDSSVEEFQTWMQHCETEMATHIPNK............
A_thaliana       KGASITPSESKFKKLIDECNFLITEEEKDLVQPVPSTLPSSV
H_vulgare        VGSSYASGDSRFTRLMKECDDRIAEEASQAPVKNAA....AA
O_sativa_Jap_    LGYSFASGDSRFTRLMKECDERIAEELSEVPVKKAE...DGA
N_benthamiana    TGASLAPAESRFTKLIKECDERIAEEAGELPNQSVDKTSGNV
```

C

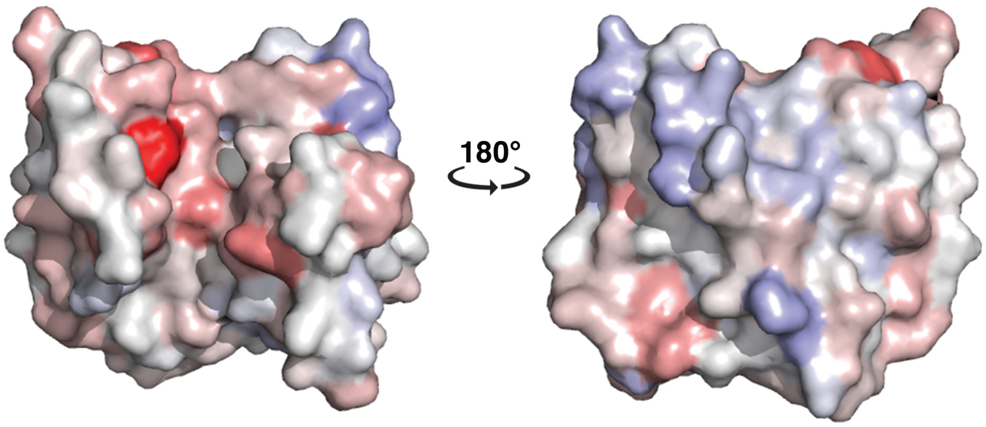

D

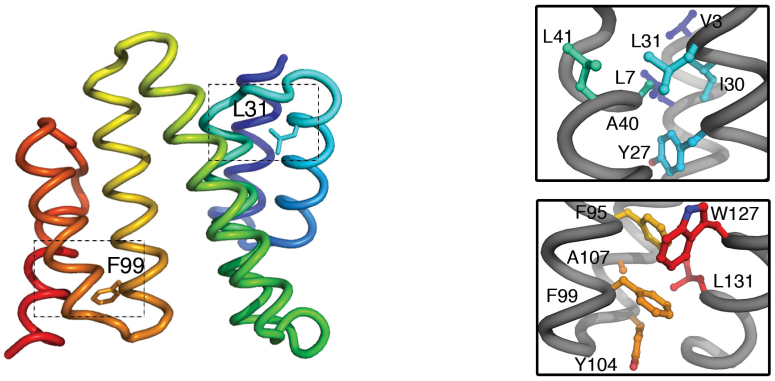

E

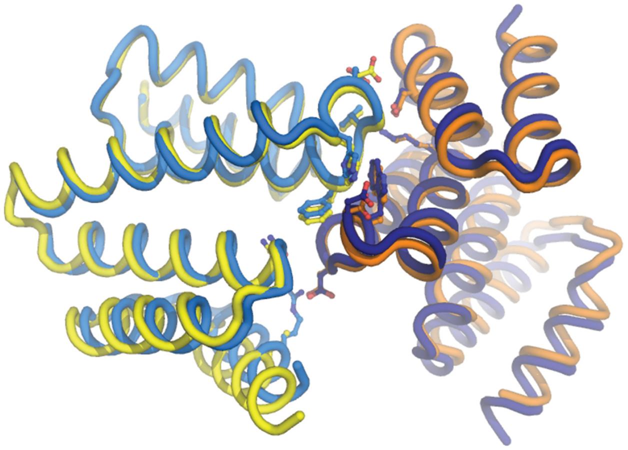

(A) RMSD fit for Cα atoms of the Sgt1TPR domain and TPR domains with high sequence identity. The PDB ID for PPP5, Sgt, CPTR3 and Hop TPR1 used for the analysis is shown, and each chain is colour-coded for clarity. **(B)** Sequence alignment of the TPR domain of homologues of Sgt1 from fungi, higher eukaryotes and plants. White text on a red background

indicates strict identity, red text indicates similarity in a group, blue frame indicates similarity across groups. Residues contributing to the oligomerization interface of the Sgt1 dimer, the Sgt1-Skp1 interaction interface, and both interfaces, are shown as blue, red and green triangles respectively. Secondary structure from the Sgt1 TPR domain is mapped to the primary structure above the alignment {Gouet:2003hk}. **(C)** Surface conservation of the TPR domain generated using a ClustalW alignment of representative eukaryotes and ProtSkin {Ritter:2004gw}. Strongly conserved:red to weakly conserved:blue. **(D)** The Sgt1TPR domain, coloured rainbow from N:blue to C:red. The mutations L31P and F99L are present in the TPR domain of Sgt1-3p. The inset boxes highlight the interactions made by these residues showing both to be within hydrophobic cores. The former mutation introduces a proline into helix 2 and will therefore disrupt the helix and consequently the structural core between helices 1, 2 and 3. Th`e latter is a conservative, non-disruptive mutation. **(E)** The two Sgt1:Sgt1 interfaces in the asymmetric unit are identical. A superposition of the BA dimer (yellow/orange) on the AC (light blue/dark blue) dimer using all atoms is shown. The RMSD of this superposition is 1.46 Å. Residues involved in the interface are shown in ball and stick.

SUPPLEMENTARY FIGURE S2: ELECTRON DENSITY MAPS AT THE SGT1-SKP1 AND SGT1-SGT1 INTERFACES

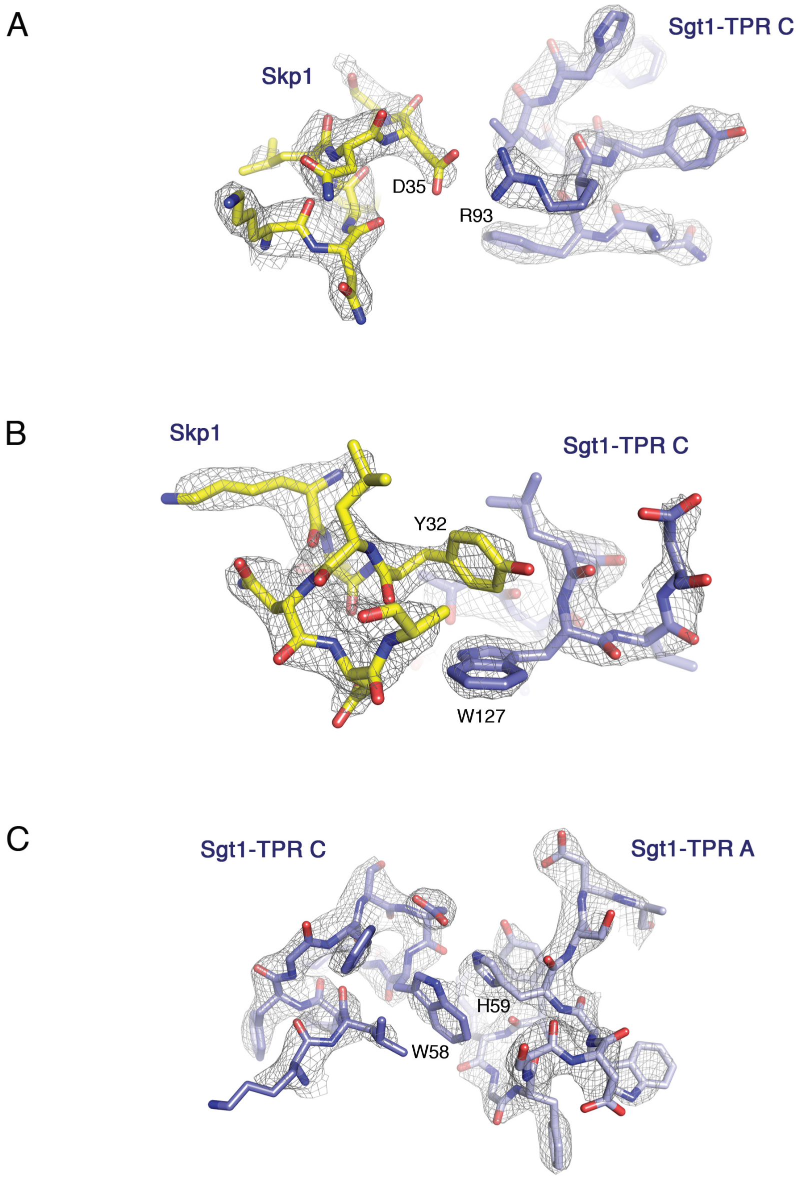

Simulated annealing composite omit maps calculated for the Sgt1-Skp1 interface at (A) Sgt1-R93 and (B) Sgt1-W127A and for the Sgt1 dimerisation interface at (C) H59.

SUPPLEMENTARY FIGURE S3: CHARACTERISATION OF SGT1 TPR DOMAIN OLIGOMERISATION

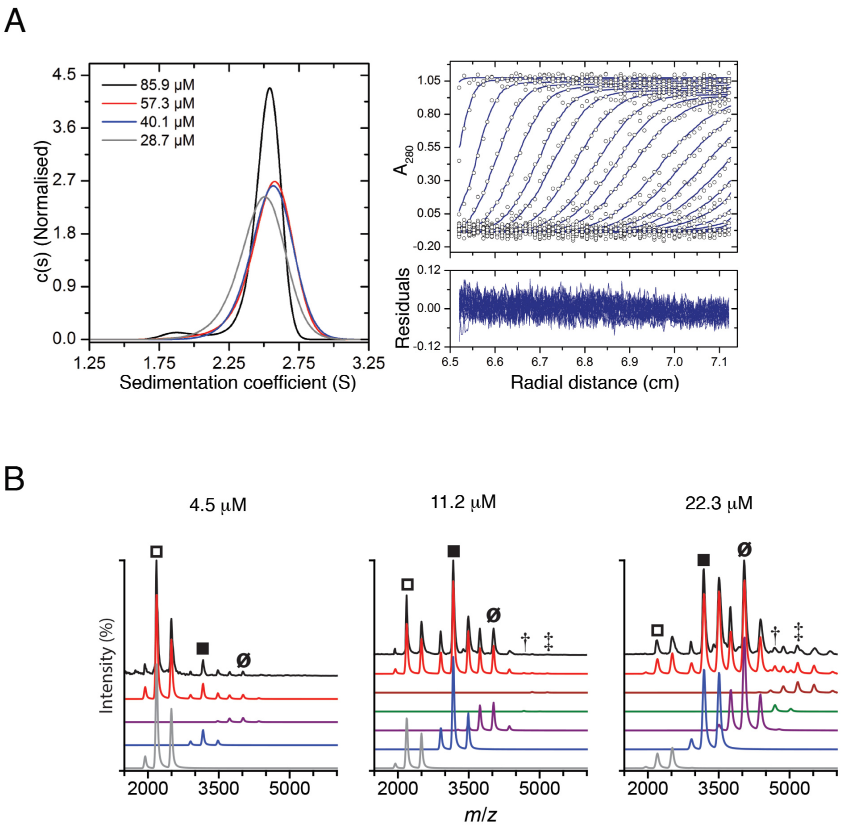

C

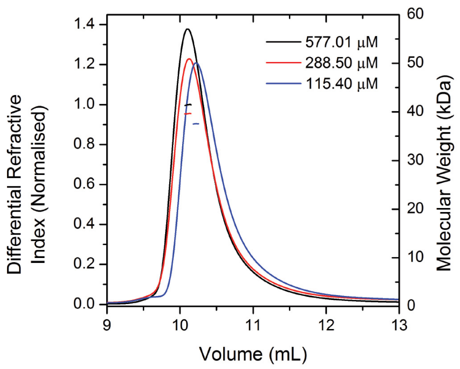

Concentration (μM)	Molar Mass (Da)
577	41450
289	39640
115	37550

(A) c(s) distributions for Sgt1TPR at 28.7 μM, 40.1 μM, 57.3 μM & 85.9 μM monomer and corresponding boundary fits and residuals for the sample generated by SV-AUC. Supplementary Table S1 details the sedimentation coefficients, MW and f/fo.

(B) Native nESI-MS spectra of Sgt1TPR at 4.5 μM, 11.2 μM and 22.3 μM. Charge state series corresponding to monomer, dimer, and trimer are indicated in grey, blue and purple

respectively. Charge states: wt +13 ○, $(wt)_2$ +20 ●, $(wt)_3$ +24 ♦. Spectra were deconvoluted to individual charge state series using Amphitrite {Sivalingam:2013vb} with the raw data shown in black and the sum of simulated spectra in red. Supplementary Table S2 details the percentage contribution of individual species at each concentration.

(C) Chromatograms and estimated molecular weights determined using a Superdex 75 HR10/30 (GE Healthcare) and multi-angle light scattering (SEC-MALS) for Sgt1TPR at 115 µM, 289 µM, 577 µM. An approximately 10-fold increase in sample concentration was used compared to the SV-AUC analysis to account for the dilution effect of size exclusion chromatography.

SUPPLEMENTARY FIGURE S4: SGT1-SKP1 IN THE CONTEXT OF TPR-MEEVD, SKP1-FBXL AND SKP1-CULLIN COMPLEXES

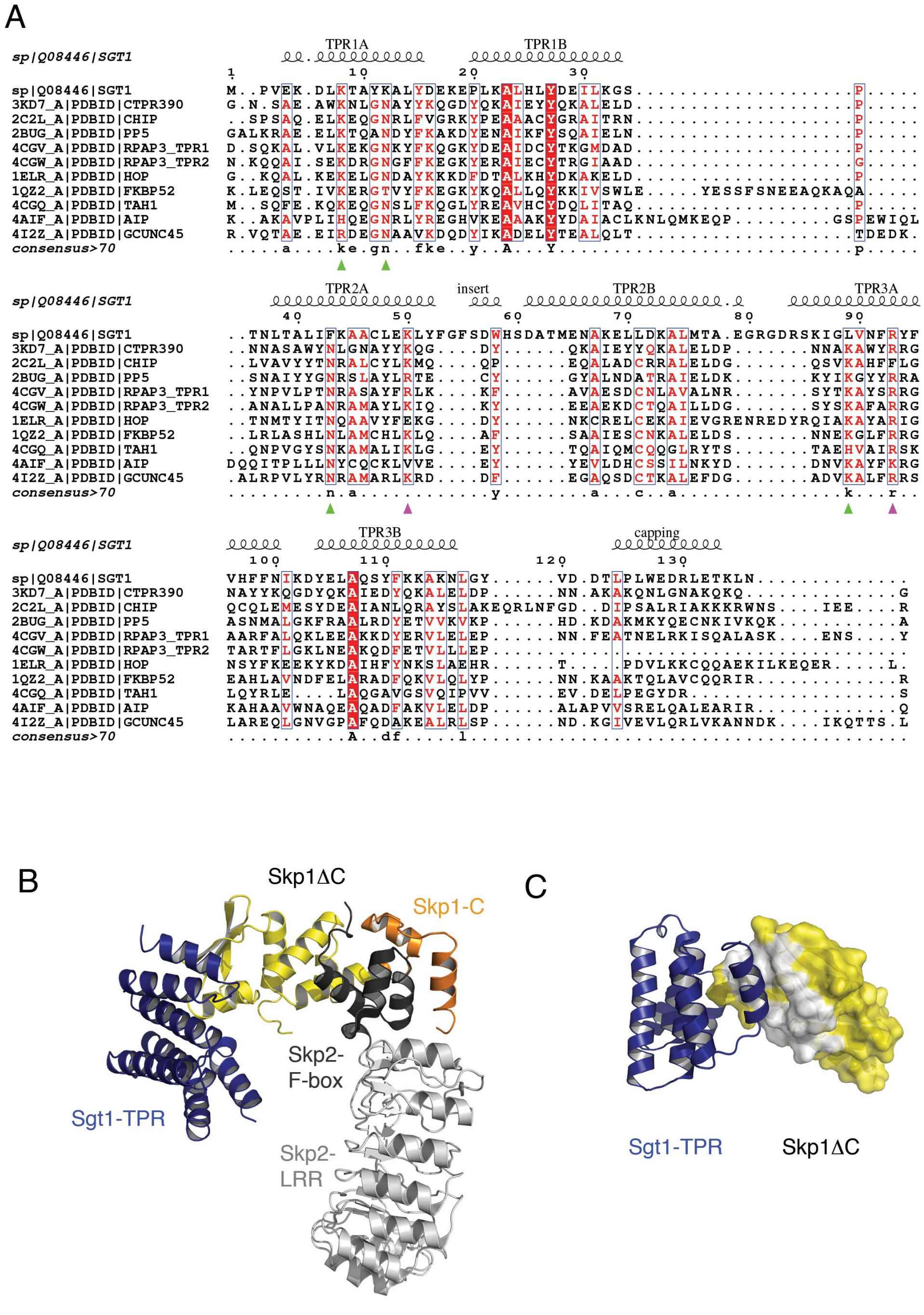

(A) A structure-based multiple sequence alignment using T-Coffee Expresso of the TPR domain of Sgt1 with TPR-cochaperones that interact with Hsp90 via its MEEVD motif with {DiTommaso:2011kx}. Cochaperones with deposited crystal structures with MEEVD interaction were selected for alignment and their PDB is labelled. Residues that contribute

conserved electrostatic interactions with the carboxylate-clamp are highlighted in green and with other residues of the MEEVD motif are highlighted in magenta.

(B) A least squares fit of the Sgt1TPR-Skp1BTBΔ crystal structure (blue and yellow respectively) with the Skp1-Skp2 heterodimer (PDB: 1FQV). The C-terminal region of Skp1 (not present in our construct) is coloured orange to distinguish it from our Skp1BTBΔ construct. The F-box and LRR domains of Skp2 are coloured different shades of grey. **(C)** The Sgt1TPR-Skp1BTBΔ crystal structure showing a surface representation of Skp1 (yellow). Residues of Skp1 that interact with Cul1 are white (from PDB 1LDK).

SUPPLEMENTARY FIGURE S5: ASSOCIATION OF SGT1 DIMERISATION MUTANTS WITH SKP1

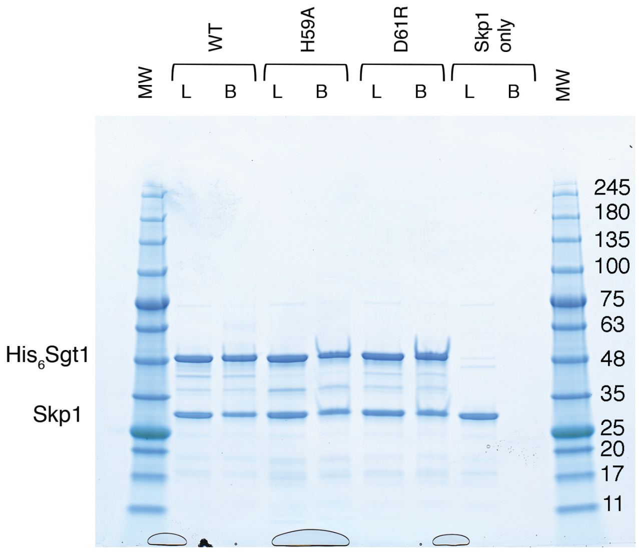

The ability of full-length Skp1 to interact with His_6-tagged full-length wild type (WT) Sgt1 and indicated mutants assessed by pull-down on Ni resin. L = 10% load, B = bound.

FIGURE S6: FULL GELS AND WESTERN BLOTS

A

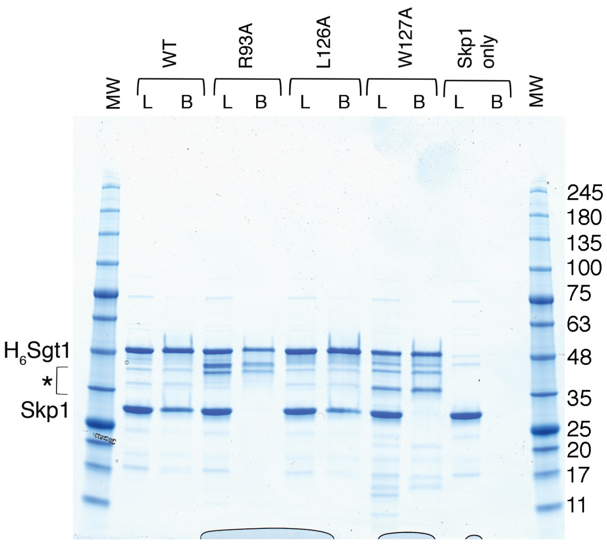

B

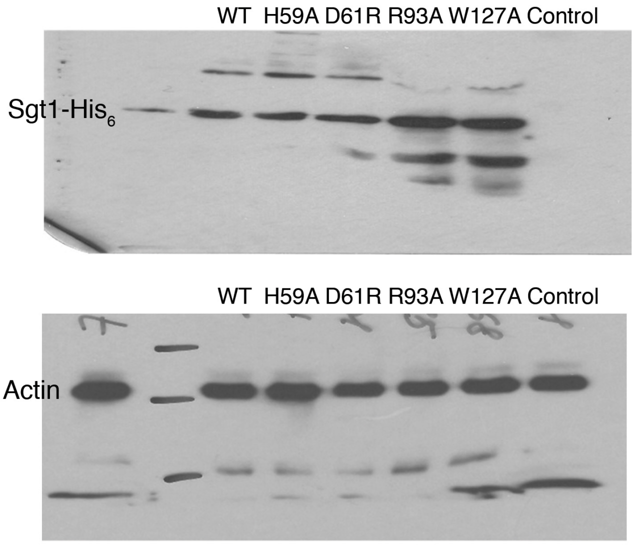

(A) Pulldown of full-length Skp1 using His_6-tagged wild type Sgt1 (WT) and indicated mutants on Ni resin. L = 10% load, B = bound. * = Degradation products of Sgt1 that copurify with the full-length protein. (B) Analysis of Sgt1 levels in JJ345 *S. cerevisiae* cultures in SD-Trp media at 28 °C expressing WT Sgt1 and either WT or mutant Sgt1-His_6. Actin was measured as a loading control.

Supplementary Tables

SUPPLEMENTARY TABLE S1: Sedimentation Velocity Analytical Ultracentrifugation for wild-type, mutant and truncation constructs of Sgt1

Protein	Concentration (μM)	s_w(20,w) (S)	Molar Mass (Da)	Frictional ratio f/f_o	% of total
WT	22.3	4.1	92158	1.78	97.6
	11.2	4.0	84097	1.70	97.6
	4.5	3.9	73891	1.60	96.1
His_6-WT	11.2	4.4	84029	1.55	92.0
His_6-H59A	11.2	2.5	41023	1.67	95.5
TPR	85.9	2.53	51031	1.797	96.5
	57.3	2.57	47597	1.733	97.6
	40.1	2.56	47644	1.752	96.8
	28.7	2.50	41116	1.583	95

SUPPLEMENTARY TABLE S2: Contribution of individual species to nESI-MS spectra of wild-type, mutant and truncation constructs of Sgt1

Protein	Concentra-tion (μM)	Monomer (%)	Dimer (%)	Trimer (%)
WT	22.3	33.1 ± 5.7	48.3 ± 2.4	18.6 ± 3.4
	11.2	19.7 ± 5.0	61.1 ± 2.1	19.2 ± 5.6
	4.5	21.9 ± 1.1	69.4 ± 7.6	8.6 ± 7.5
His_6-H59A	11.2	71.4 ± 4.9	28.6 ± 4.9	-
His_6-D61R	11.2	22.1 ± 7.0	72.6 ± 6.0	5.3 ± 3.6
TPR	22.3	10.4 ± 1.8	33.8 ± 4.6	33.7 ± 6.5*
	11.2	25.7 ± 4.6	45.9 ± 2.1	26.8 ± 4.9
	4.5	80.0 ± 4.9	13.8 ± 2.1	6.3 ± 4.0

Errors are representative of 3 replicates for H59A and 4 replicates for WT and D61R.

* This concentration of Sgt1TPR also has contributions from tetramer (7.3 ± 4.1%) and pentamer (10.1 ± 4.6%).

SUPPLEMENTARY TABLE S3: Crystallography statistics

**Data collection**	**SAD**
Space Group	**P 3_2 2 1**
Wavelength (Å)	**0.979494**
Unit cell *a*, *b*, *c* (Å)	**94.6, 94.6, 123.0**
Resolution (Å)	**81.9 – 2.8 (2.9 - 2.8)**
Total observations	**339894**
Total unique obs.	**15826**
R_{merge}	**0.089 (0.826)**
Completeness (%)	**100.0 (100.0)**
Anom. Comp. (%)	**100.0 (99.9)**
Multiplicity	**21.5 (21.9)**
Anomalous multiplicity	**11.5 (11.4)**
$I/\sigma I$	**29.2 (4.9)**
**Refinement**	
R_{work}/R_{Free}	**20.10%/23.92%**
Number of protein atoms	**3707**
r.m.s.d. bond lengths (Å)	**0.010**
r.m.s.d. bond angles (°)	**0.980**
Ramachandran plot	
Preferred region	**98.37%**
Allowed	**1.63%**
Outliers	**0%**
